# Integrated microRNA and mRNA Signature Associated with the Transition from the Locally Confined to the Metastasized Clear Cell Renal Cell Carcinoma Exemplified by miR-146-5p

**DOI:** 10.1371/journal.pone.0148746

**Published:** 2016-02-09

**Authors:** Zofia Wotschofsky, Linda Gummlich, Julia Liep, Carsten Stephan, Ergin Kilic, Klaus Jung, Jean-Noel Billaud, Hellmuth-Alexander Meyer

**Affiliations:** 1 Department of Urology, Charité - Universitätsmedizin Berlin, Berlin, Germany; 2 Berlin Institute for Urologic Research, Berlin, Germany; 3 Department of General, Visceral, Vascular and Thoracic Surgery, Division of Molecular Biology, Charité - Universitätsmedizin Berlin, Berlin, Germany; 4 Institute of Pathology, Charité - Universitätsmedizin Berlin, Berlin, Germany; 5 QIAGEN, 1700 Seaport Blvd, Redwood City, California, United States of America; The University of Hong Kong, CHINA

## Abstract

**Background:**

MicroRNAs (miRNAs) regulate gene expression by interfering translation or stability of target transcripts. This interplay between miRNA and their mRNA has been proposed as an important process in cancer development and progression. We have investigated molecular networks impacted by predicted mRNA targets of differentially expressed miRNAs in patients with clear cell renal cell carcinoma (ccRCC) diagnosed with or without metastasis.

**Material and Methods:**

miRNA and mRNA microarray expression profiles derived from primary ccRCC from patients with (16 samples) or without diagnosed metastasis (22 samples) were used to identify anti-correlated miRNA-mRNA interaction in ccRCC. For this purpose, Ingenuity pathway analysis microRNA Target Filter, which enables prioritization of experimentally validated and predicted mRNA targets was used. By applying an expression pairing tool, the analysis was focused on targets exhibiting altered expression in our analysis, finding miRNAs and their target genes with opposite or same expression. The resulting identified interactions were revalidated by RT-qPCR in another cohort of ccRCC patients. A selection of the predicted miRNA-mRNA interactions was tested by functional analyses using miRNA knockdown and overexpression experiments in renal cancer cell lines.

**Results:**

Among the significantly differentially expressed miRNAs, we have identified three miRNAs (miR-146a-5p, miR-128a-3p, and miR-17-5p) that were upregulated in primary tumors from patients without metastasis and downregulated in primary tumors from patients with metastasis. We have further identified mRNA targets, which expression were inversely correlated to these 3 miRNAs, and have been previously experimentally demonstrated in cancer setting in humans. Specifically, we showed that CXCL8/IL8, UHRF1, MCM10, and CDKN3 were downregulated and targeted by miR-146a-5p. The interaction between miR-146a-5p and their targets CXCL8 and UHRF1 was validated in cell culture experiments.

**Conclusions:**

We identified novel target genes of dysregulated miRNAs, which are involved in the transition from primary RCC without metastases into tumors generating distant metastasis.

## Introduction

Renal cell carcinoma (RCC) is the most common type of kidney tumor in adults. In United States, 61560 new cases of RCC are expected in 2015 and almost 30% of these patients will eventually succumb to their disease in the next 5 years [1]. In general, primary RCC shows no typical early clinical symptoms, therefore the tumor is most often first discovered by a routine ultrasonic investigations. At this time point, approximately 20% of the patients already show distant metastases and another 30% of the patients will develop metastases after radical nephrectomy [2]. There are three main different morphotypes of RCC [3]. Clear cell RCC (ccRCC) accounts for approximately 80–90% of all RCCs, while 6–15% and 2–5%, respectively are papillary RCCs and chromophobe RCCs. Both this high frequency of ccRCC but also the significantly worse clinical outcome for patients with ccRCC after nephrectomy compared with patients suffering from papillary or chromophobe RCC determine the clinical significance of ccRCC [3]. The standard treatment of localized RCC is the radical tumor nephrectomy, whereas in recent years a nephron sparing surgery has emerged as a safe alternative for small primary tumors [4]. In case of metastatic RCC, the mainly infested organs are lungs, bones, liver and brain, which cause a high morbidity and a poor prognosis. At the moment, there is no general effective curative treatment for metastatic RCC. Nevertheless, considerable progress has been made due to the introduction of individualized therapies by using tyrosine kinase inhibitors and angiogenesis inhibitors [5]. In the case of transition from a primary RCC without metastases into a tumor generating distant metastasis, there is a clear need for novel prognostic biomarkers to ensure adequate risk stratification and to help with the choice of therapy options [6].

MicroRNAs (miRNAs) play a key role in gene regulation, they are now being explored to identify potential disease biomarkers and new targets [7]. Several miRNA and mRNA expression studies have been conducted to characterize the molecular mechanisms of ccRCC development [7] and a genome atlas of this tumor was recently established [8]. To get a deeper insight into the process of tumor transition from primary ccRCC into a tumor which is capable to generating distant metastases an integrated analysis of both miRNA and mRNA expression data is advisable [9–12]. The data from our previous miRNA expression studies of ccRCC tissue samples showed that progression from non-metastatic to metastatic tumor was not always reflected by a continuous process of molecular changes in a straight line [13,14]. We observed different expression shifts in this transition process, surely a consequence of the multiple interactions between the various molecular cellular components. Thus, we believe that the study of molecular alterations in the primary tumor could better characterize this transition process from non-metastatic to the metastatic tumor than examination of metastatic lesions.

Unfortunately, searching miRNA databases for predicted and observed mRNAs yields thousands of potential targets [10,15], and this is a significant challenge for identifying and classifying the truly relevant targets. Moreover, the inherent variability in experimental samples, technology platforms, and analysis methods makes finding targets that translate well to biomarkers that much more difficult. Therefore, integrated tools were needed to analysis miRNA and mRNA data as well as involved biological relationships in one process. For this purpose, Ingenuity Pathway Analysis (IPA^®^) was used (QIAGEN Redwood City, CA, www.qiagen.com/ingenuity). The IPA^®^’s microRNA Target Filter enables to go directly from preprocessed miRNA expression data to zeroing in on the most promising mRNA targets by using known biological evidence around molecular interactions and disease mechanisms whether or not we have mRNA measurements from the same sample.

Thus, the aim of our study was to use the IPA^®^’s MicroRNA Target Filter platform to explore new interactions between miRNAs and mRNAs in ccRCC for detecting novel biomarkers or targets. The usefulness of this tool in this promising research concept could be demonstrated by the example of miR-146a-5p and its potential targets chemokine (C-X-C motif) ligand 8/interleukin 8 (CXCL8; alias IL8), ubiquitin-like with PHD and ring finger domains 1 (UHRF1), breast cancer 1, early onset (BRCA1), minichromosome maintenance complex component 10 (MCM10), and cyclin-dependent kinase inhibitor 3 (CDKN3). The experimental validation of this approach was shown by means of the modulation of the expression of CXCL8 and UHRF1 on mRNA and protein level in cell culture experiments.

## Materials and Methods

### Patients and tissue samples

The ccRCC samples were obtained during partial or radical nephrectomy at the University Hospital Charité in Berlin between 2004 and 2008. The study was approved by the Ethic Committee of the University Hospital Charité (EA1/153/07 and EA1/153/12: "microRNAs as diagnostic and prognostic signatures in urological tumors"). The study was conducted in compliance with the declaration of Helsinki and written informed consent has been obtained. The staging and grading of the tumor samples were classified according to the 2002 TNM classification and the Fuhrman grading system [16,17]. All tissue samples were frozen in liquid nitrogen directly after surgical resection and stored at -80°C until RNA extraction.

miRNA microarray expression profiles were generated from a selection of 24 matched malignant and non-malignant kidney tissue samples from a collective of ccRCC patients described previously [13]. In detail, samples from 8 patients without diagnosed metastasis (ccRCC-M0; 5 male and 3 female patients; median age 67, range 39–73 years; tumor staging: 1x pT1, 1x pT2, and 6x pT3; grading: 6x G2 and 2x G3) and from 4 patients with diagnosed metastasis (ccRCC-M1; 2 male and 2 female patients; median age 64.5, range 57–74 years; tumor staging: 4x pT3; grading: 1x G1 and 3x G2) were analyzed.

mRNA microarray expression analysis was performed by using two different collectives of RCC patients. The first set contained 28 matched malignant and non-malignant kidney tissue samples from 14 ccRCC-M0 patients (11 male and 3 female patients; median age 66, range 45–78 years; tumor staging: 10x pT1, 1x pT2, and 3x pT3; grading: 1x G1 and 13x G2). The second set consisted of 26 matched malignant and non-malignant kidney tissue samples from 13 ccRCC-M1 patients (8 male and 5 female patients; median age 62, range 40–75 years; tumor staging: 2x pT1, 10x pT3, and 1x pT4; grading: 3x G2; 8x G4, and 2x G4).

For the quantitative real-time reverse-transcription PCR (RT-qPCR) analysis, another set of patients was used, containing 10 non-malignant kidney tissue samples of ccRCC patients (median age 58, range 41–75), 10 tissue samples of primary ccRCC from ccRCC-M0 patients (9 male and 1 female patients; median age 67, range 39–73 years; tumor staging: 4x pT1a, 3x pT1b, 1x pT2, and 2x pT3a; grading: 2x G1 and 8x G2) and 10 tissue samples of primary ccRCC from ccRCC-M1 patients (6 male and 4 female patients; median age 60.5, range 40–74 years; tumor staging: 3x pT3a, 4x pT3b, 1x pT3c, and 2x pT4; grading: 4x G2, 5x G3, and 1x G4).

### miRNA microarray expression analysis

miRNA expression analysis was performed as described before with one-color hybridizations on human catalog 8-plex 15 K microRNA microarrays (AMADID 016436; Agilent Technologies, Santa Clara, CA, USA) encoding probes for 470 human and 64 human viral miRNAs from the Sanger database v9.1 [13,18]. After scanning, features were extracted with the image analysis tool version A.9.5.3 using default protocols and settings (Agilent Technologies). The raw scan data were interpreted using Genespring GX11 Software (Agilent Technologies) with default input parameters (threshold raw signal to 1.0, percent shift to 90th percentile as normalization algorithm and no baseline transformation). The corresponding expression data were archived under the GEO Accession No. GSE37989.

### mRNA microarray expression analysis

mRNA expression analysis was performed by one-color hybridizations on Human Genome U133 Plus 2.0 Arrays (Affymetrix, Santa Clara, CA, USA). After hybridization, microarrays were washed, scanned, and processed according to the supplier's protocol (Affymetrix). The raw data were normalized using Genespring GX11 Software (Agilent Technologies) with default parameters (MAS5 Summarization Algorithm, median of all samples as baseline transformation). The two sets of primary tumor samples (M0 and M1) were analyzed separately. The corresponding expression data were archived under the GEO Accession No. GSE66272 and GSE66271.

### RNA extraction and RT-qPCR analysis

Total RNA, including miRNAs was extracted from archived frozen ccRCC tissue histologically verified and renal cancer cell lines using the miRNeasy Mini Kit (QIAGEN, Hilden, Germany) according to the instructions provided by the manufacturer and previously described [13,18] (S1 Fig). The adjacent normal tissue was selected at a distance of >20 mm to the cancer tissue to avoid possible alterations of the non-neoplastic tissue through the tumor. Total RNA quantity was determined on a NanoDrop 1000 Spectrometer (Thermo Fisher Scientific Inc., NanoDrop products, Wilmington, DE, USA) by calculation of A260/230 and A260/280 ratios and the quality of the RNA was investigated using a Bioanalyzer 2100 (Agilent Technologies) with an RNA 6000 Nano Lab Chip. Only samples with RNA integrity number values above 6 and ratios above 1.8 were included into the analysis.

For mRNA quantification, complementary DNA synthesis was performed using the Transcriptor First Strand cDNA Synthesis Kit (Roche Applied Science, Mannheim, Germany). The relative quantification of transcripts was done on the Light Cycler 480 (Roche Applied Science) using the QuantiTec SYBR Green PCR Kit as previously described [19–21]. Briefly, 1 μg total RNA was reverse transcribed in a total volume of 20 μl. For the PCR reactions, 1 μl of cDNA was amplified using 2.5 μM transcript-specific primers (TIB Molbiol, Berlin, Germany) in a final volume of 12.5 μl. The reactions were performed at 95°C for 15 min, followed by 45 cycles with denaturation at 94°C for 15 s, variable primer annealing temperature for 30 s (S1 Table) and elongation at 72°C for 30 s. The samples were measured in triplicates and non-template control and interplate controls were included in each PCR run.

The TaqMan MicroRNA primer assays (Life Technologies GmbH, Applied Biosystems, Darmstadt, Germany) were used for determination of mature miRNA in accordance to the manufactures protocol and MIQE guidelines [22] and previously described [18,19,23]. cDNA was synthesized with 6.67 ng of total RNA using TaqMan MicroRNA reverse transcription Kit (Life Technologies) and miRNA-specific stem-looped primers in a total volume of 10 μl. PCR measurements were performed on the Light Cycler 480 Instrument (Roche) using 1 μl cDNA, 1x TaqMan Universal PCR Master Mix, No AmpErase UNG and miRNA-specific primers in a total volume of 10 μl in according to the manufacturer's recommendations.

PCR data were analyzed by GenEX software (MultiD Analyses AB, Göteborg, Sweden) or qBase^PLUS^ software (Biogazelle NV, Gent, Belgium) using the correction of amplification efficiencies and the interplate variance. The miRNA expression data were normalized to the reference gene combination of miR-28, miR-103, and miR-106a [18]. The mRNA expression data were normalized to the reference gene peptidylproline isomerase A (PPIA) [19]. The suitability of both normalization approaches using the mentioned reference genes was confirmed for this study (S2 Fig).

### Cell culture

Human kidney cancer cell lines 786-O and ACHN were used (American Type Culture Collection, Manassas, VA, USA). 786-O cells were maintained in RPMI 1640 (Life Technologies GmbH, Invitrogen, Darmstadt, Germany) and ACHN cells were cultured in Eagle's Minimum Essential Medium (Biochrom GmbH, Berlin, Germany). The media were supplemented with 10% fetal calf serum (PAA Laboratories, Pasching, Austria) and 1% penicillin-streptomycin (PAA Laboratories) and both cell lines were grown in a humidified 5% CO_2_ incubator at 37°C.

For transfection, 0.8x10^5^ 786-O cells or 2x10^5^ ACHN cells per well were seeded into 6-well plates. The next day, the cells were transfected with 30 nM mimic miR-146a, 30 nM mimic negative control 1 (NC1), and 50 nM inhibitor miR-146a (Life Technologies GmbH, Ambion, Darmstadt, Germany) using Lipofectamine2000 Reagent (Invitrogen). After 48 h incubation, the supernatant was collected for CXCL8 ELISA assay, the cells were lysed in lysis buffer (0.5 mM Tris pH 6.8, 1% SDS, 1 mM EDTA, 1 mM PMSF, 100 μg/ml Trypsin Inhibitor, 10 μg/ml Aprotinin), and protein amount was determined for Western blot analysis.

### CXCL8 (IL8) ELISA assay

Media supernatant interleukin-8 (CXCL8, alias IL8) protein levels were measured using a solid phase sandwich Elisa (Human CXCL8 DuoSet ELISA kit; DY208-05, R&D Systems Inc., Minneapolis, MN, USA). A calibration curve was prepared with CXCL8 standards of 31.3, 62.5, 75, 125, 150, 250, 300, 500, and 1000 pg/ml. Capture antibody (1:120), detection antibody (1:60), and streptavidin (1:40) were applied using an optimized protocol. After incubation of the substrate solution for 10 min, the reaction was stopped and measured at 450 nm (reference 540 nm). The experiment was performed three times in duplicates and CXCL8 production normalized to cell number.

### UHRF1 Western blot analysis

A total of 20 μg of protein per gel pocket were separated by SDS-PAGE (90 V for 15 min, 130 V for 1.5 h) before Western blotting (120 mA, overnight, 4°C). The membrane was blocked in a 5% milk/PBS-T solution at RT for 2 h before application of the first antibodies rabbit-UHRF1 (1:200, #12387; Cell Signaling Technology, Danvers, MA, USA) and mouse-γ-tubulin (1:1000, sc-7396, Santa Cruz Biotechnology, Dallas, TX, USA) over night at 4°C. The next day, secondary antibodies were applied (anti-rabbit/anti-mouse, 1:1000, Seramun Diagnostica GmbH, Heidesee, Germany) for 2 h at RT and developed using enhanced chemiluminescence method (Pierce Biotechnology, Rockford, IL, USA). The experiment was repeated three times and densitometric analysis was performed.

### Data analysis and statistics

miRNA-mRNA interactions were analyzed using the Ingenuity Pathway Analysis (IPA^®^) (QIAGEN Redwood City, CA, www.qiagen.com/ingenuity) as mentioned. In detail, for the classification of the miRNA-mRNA interactions differentially expressed miRNAs were associated to experimentally validated and predicted mRNA targets from TarBase, miRecords, Target Scan, and the Ingenuity® Knowledge Base. The resulting miRNA-mRNA interactions pairs were mapped with the previously identified differentially expressed mRNAs. The resulting interaction networks of differentially expressed miRNAs and mRNAs were visualized by IPA^®^.

Statistical analysis data were performed with GraphPad Prism 6.07. (GraphPad Software, San Diego, CA, USA) and MedCalc 15.8 (MedCalc Software bvba, Ostend, Belgium). The non-parametric Mann-Whitney U-test and the parametric Student’s t-test with log-transformed data were used to analyze significant differences between the groups. All tests were performed two-tailed and p<0.05 values were considered statistically significant in all cases.

## Results

### Identification of miRNA target genes using IPA^®^'s MicroRNA Target Filter

In order to identify new miRNA target gene interactions, which are involved in the process of tumor transition, we analyzed miRNA and mRNA expression profiles from malignant and non-malignant kidney tissue samples from patients with or without diagnosed metastasis. Using IPA^®^'s MicroRNA Target Filter analysis we found 54 differentially regulated miRNAs in the processed datasets of normal and ccRCC samples. The 54 identified miRNAs, including associated miRNA families, target 13491 possible mRNAs. These either experimentally observed or predicted miRNA-mRNA associations were sourced from TarBase, miRecords, Target Scan, and the Ingenuity^®^ Knowledge Base.

**Fig 1:**
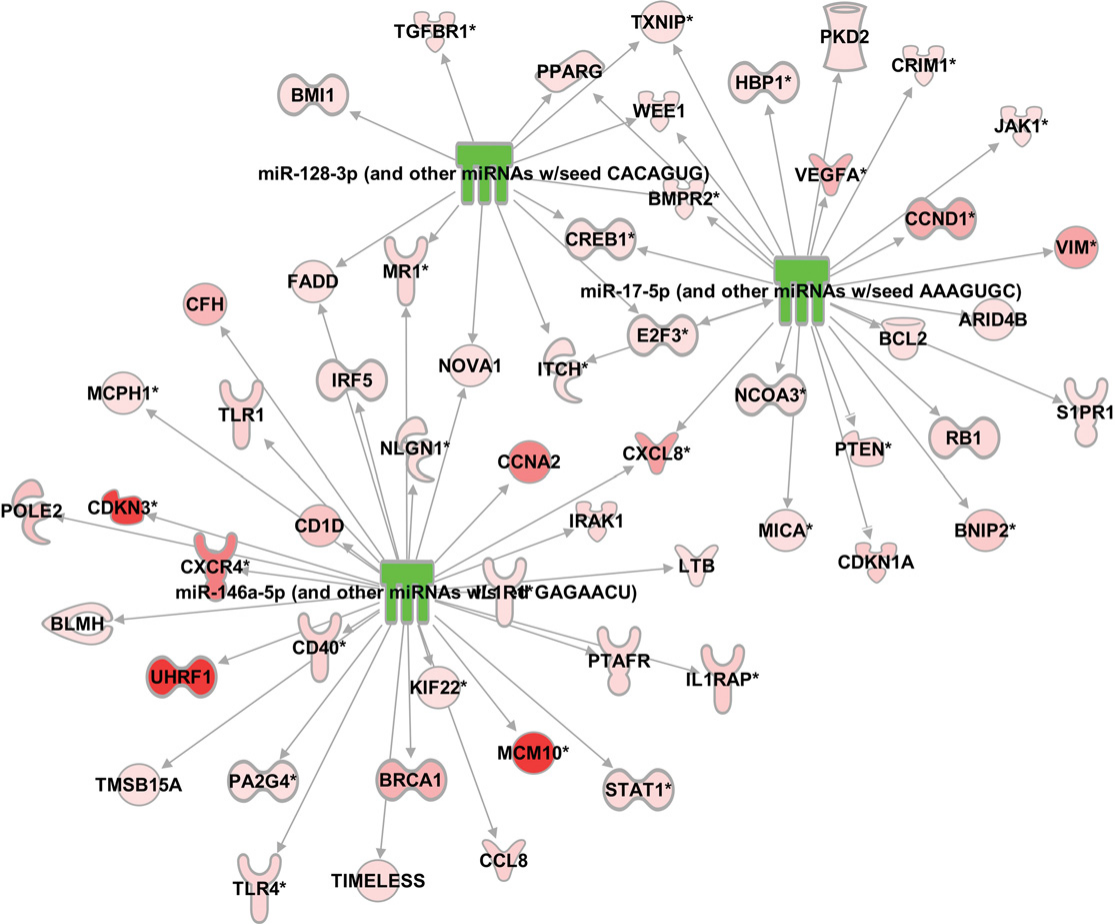
Interaction Network between 3 differentially expressed miRNAs (downregulated M1 vs M0, green) and differentially expressed mRNAs (upregulated M1 vs M0, red) from ccRCC patients.

In the next filtering step, we selected only experimentally observed miRNA-mRNA interactions based on inverse expression pairings in our datasets. Subsequently, we focused only on observed relationships between miRNAs and mRNAs in the human species and relationships observed in cancer only. Finally, we obtained a group of 32 miRNAs that associate with 348 mRNAs (S2 Table). Among these significantly differentially expressed 32 miRNAs, IPA^®^'s MicroRNA Target Filter identified three miRNAs (miR-146a-5p, miR-128a-3p, and miR-17-5p) that were upregulated in primary tumors from ccRCC-M0 patients and only downregulated in primary tumors from ccRCC-M1 patients (Fig 1).

### Expression of selected miRNAs in non-malignant, ccRCC-M0, and ccRCC-M1 samples

**Fig 2:**
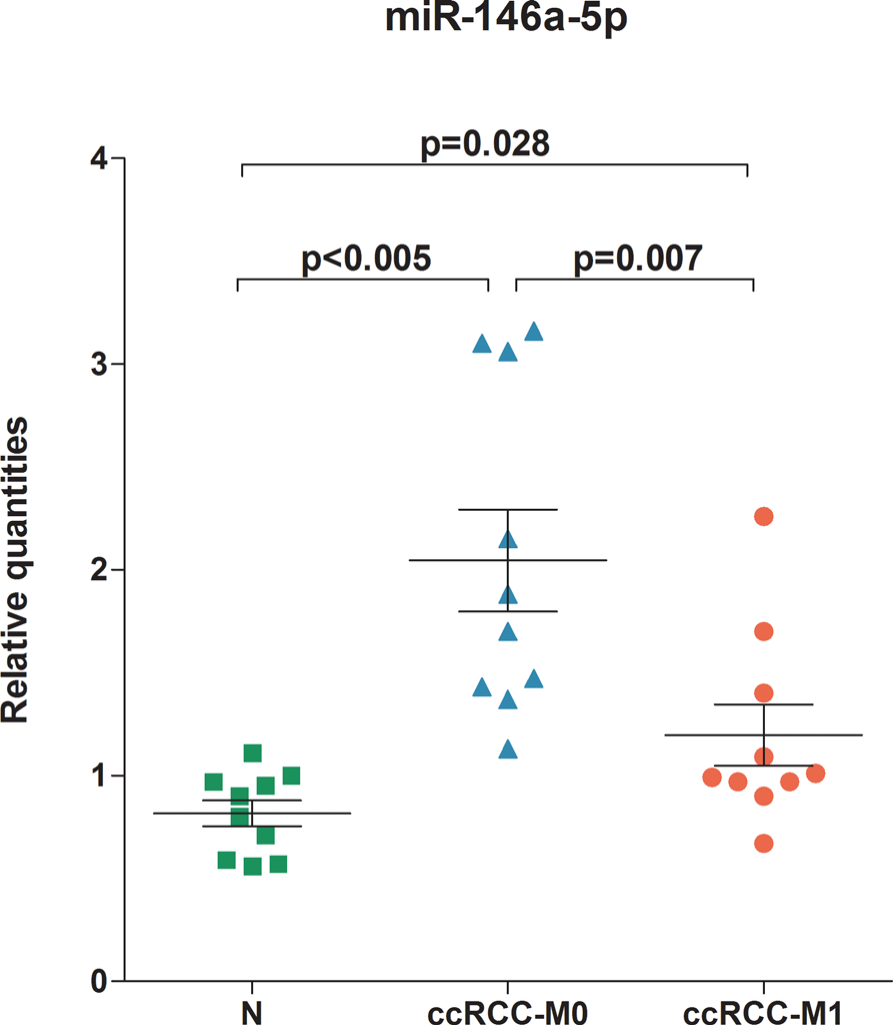
Expression of miR-146a-5p in ccRCC tissue samples. RT-qPCR analysis of the expression of miR-146a-5p in ccRCC tissue samples was performed. The data are represented in a scatter dot plots with means for normal, non-malignant renal tissue samples (N; n = 10), primary tumor samples without diagnosed metastasis (ccRCC-M0; n = 10), and primary tumor samples with diagnosed metastasis (ccRCC-M1; n = 10). Normalization was assessed with the reference miRNA combination miR-28, miR-103, and miR-106a [18], (S2 Fig). Statistical differences were calculated using the Mann-Whitney U test between the groups.

To confirm the results of the IPA^®^'s MicroRNA Target Filter analysis, the three miRNAs miR-146a-5p, miR-128-3p, and miR-17-5p were revalidated by RT-qPCR in a second set of samples, including normal kidney and primary ccRCC samples from ccRCC-M0 and ccRCC-M1 patients. miR-17-5p and miR-128-3p showed no significant differences in expression between the three sample groups, whereas miR-146a-5p was significantly higher expressed in the tested ccRCC-M0 and ccRCC-M1 tissues in comparison to the normal kidney (Table 1). More importantly, miR-146a-5p is significantly lower expressed in metastatic primary ccRCC tumors (ccRCC-M1) in comparison to non-metastatic primary ccRCC tumors (ccRCC-M0) (Fig 2). It is of interest that miR-146a-5p shares the same seed sequence (GAGAACU) with the miR-146b-5p, and in IPA^®^, both miRNAs are associated in the same family mir-146. However, miR-146b-5p was only differentially expressed between non-malignant tissue and ccRCC-M0 or ccRCC-M1 tissue samples but not between the two tumor tissues ccRCC-M0 and ccRCC-M1 (Table 1). In consequence of these results, our further interest was only focused on the potential targets of miR-146a-5p.

**Table 1:** Expression of selected miRNAs in non-malignant, ccRCC-M0 and ccRCC-M1 samples.

miRNA	ccRCC-M0 to Normal^a^		ccRCC-M1 to ccRCC-M0^a^		ccRCC-M1 to Normal^a^	
	Folds (mean ± SD)					
		*p-*value^b^		*p-*value^b^		*p-*value^b^
**miR-17-5p**	2.56 ± 0.70 to 2.13 ± 0.35	0.241	2.79 ± 0.97 to 2.56 ± 0.70	0.762	2.79 ± 0.97 to 2.13 ± 0.35	0.104
**miR-128a-3p**	0.38 ± 0.27 to 0.27 ± 0.06	0.937	0.34 ± 0.16 to 0.38 ± 0.27	0.762	0.34 ± 0.16 to 0.27 ± 0.06	0.649
**miR-146a-5p**	2.05 ± 0.79 to 0.82 ± 0.2	<0.001	1.2 ± 0.47 to 2.05 ± 0.79	0.007	1.2 ± 0.47 to 0.82 ± 0.2	0.028
**miR-146b-5p**	1.96 ± 1.13 to 0.81 ± 0.35	0.005	2.11 ± 1.52 to 1.96 ± 1.13	1.000	2.11 ± 1.52 to 0.81 ± 0.35	0.019

^a^Quantitative RT-PCR analysis of miR-17-5p, miR-128a, and miR-146a-5p was performed in different tissue samples: normal, non-malignant renal tissue (n = 10, Normal), primary ccRCC samples without diagnosed metastasis (n = 10, ccRCC-M0), and primary ccRCC samples with diagnosed metastasis (n = 10, ccRCC-M1). Normalization was assessed with the reference miRNA combination miR-28, miR-103, and miR-106a [18], (S2 Fig).

^b^Statistical differences were calculated using the Mann-Whitney U test between tissue groups (Normal, ccRCC-M0, and ccRCC-M1) for each miRNA.

### Selection of mRNAs targeted by miR-146a-5p

Using IPA^® '^s MicroRNA Target Filter analysis we obtained a set of 32 mRNA targets, which expression were inversely correlated to the miR-146a-5p expression (Fig 1). In ccRCC-M1, the expression of miR-146a-5p is significantly downregulated in comparison to ccRCC-M0. Therefore, we were interested in mRNA targets out of the group of 32 which were highly upregulated in ccRCC-M1 (M1 vs normal) and lower expressed in ccRCC-M0 (M0 vs normal). For further exploration, we selected five genes that have been previously experimentally demonstrated in other cancer setting in humans, *CXCL8/IL8*, *UHRF1*, *BRCA1*, *MCM10*, and *CDKN3* (S3 Fig).

### Expression of target mRNAs in non-malignant, ccRCC-M0 and ccRCC-M1 samples

**Fig 3:**
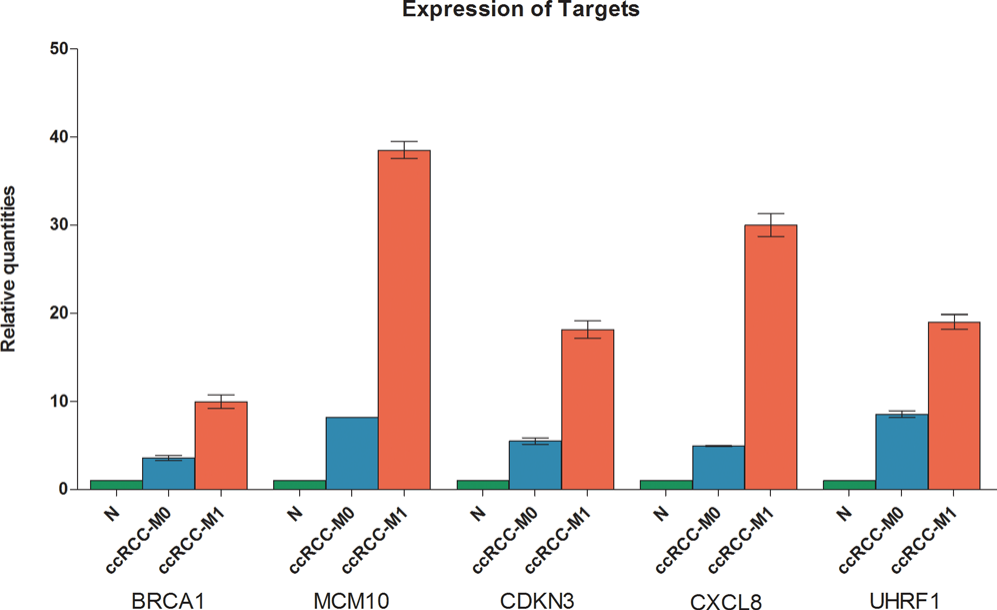
Expression of putative mRNAs targeted of miR-146a-5p. The relative mRNA expression levels of the potential miR-146a-5p targets BRCA1, MCM10, CDKN3, CXCL8/IL8, and UHRF1 were measured in duplicates in a pool of normal renal tissue and tissue samples from primary ccRCC-M0 and ccRCC-M1 patients by RT-qPCR. Data were normalized with PPIA reference gene [19], (S2 Fig). BRCA1, CDKN3, MCM10, CXCL8/IL8, and UHRF1 are lower in tissue samples of ccRCC without metastasis compared to ccRCC with metastasis.

The expression of the selected mRNA targets (CXCL8, UHRF1, BRCA1, MCM10, and CDKN3) were analyzed in pool samples from collectives of normal kidney, primary ccRCC-M0 and primary ccRCC-M1 tissues by RT-qPCR. An upregulation of mRNA expression was detected in all five tested target mRNAs in ccRCC-M1 samples in comparison to ccRCC-M0 samples and normal kidney samples (Fig 3).

### Experimental validation of mRNA-miR-146a-5p interaction in cancer cell lines

**Fig 4:**
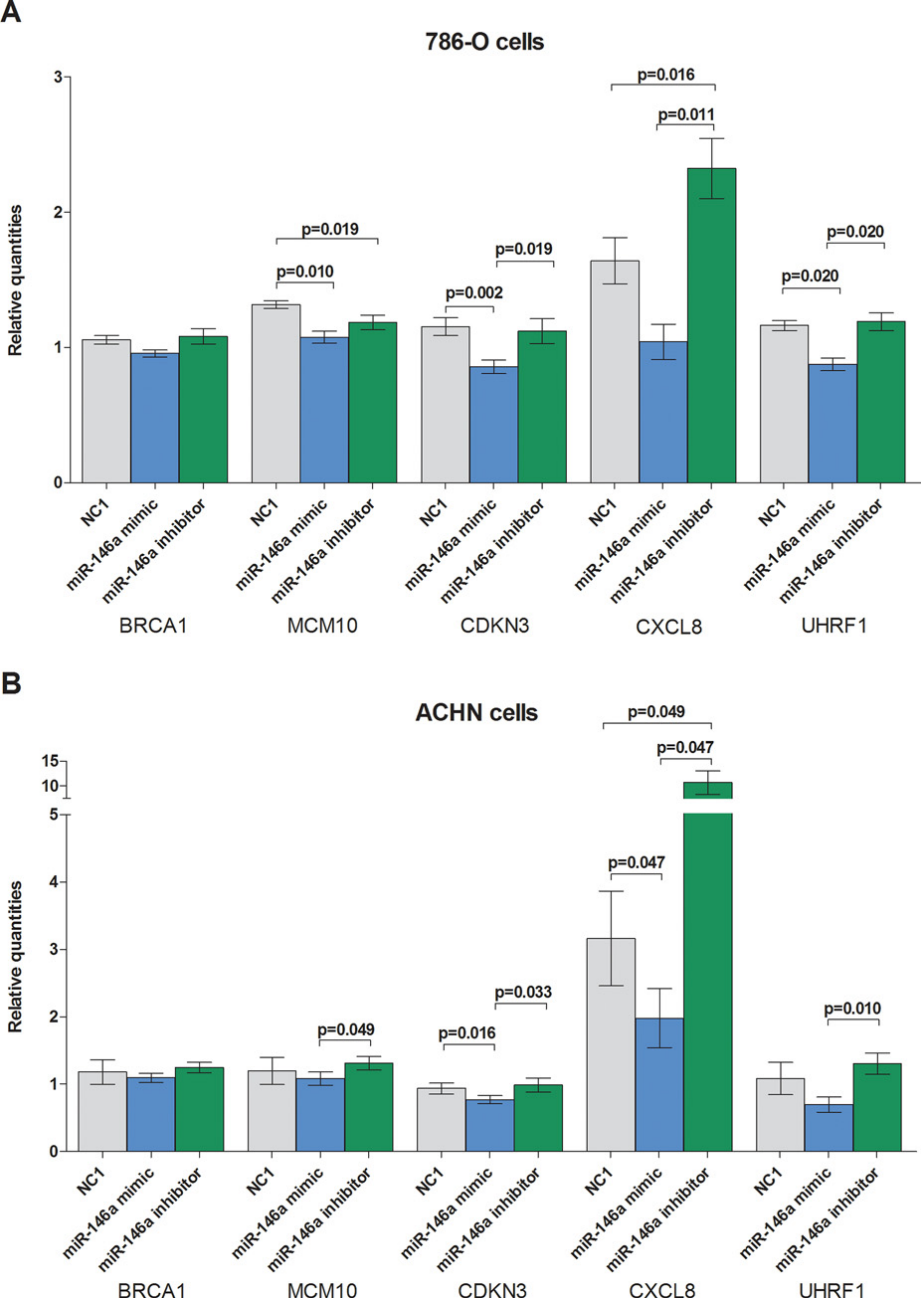
Effect of miR-146a-5p on putative mRNA target levels in 786-O and ACHN cells. Application of miR-146a-5p mimic and miR-146a-5p inhibitor effects mRNA target expression in cell lines. Data were normalized with PPIA reference gene [19]. NC1 = negative control.

As proof of concept, we used the two different human kidney cancer cell lines, 786-O derived from primary ccRCC and ACHN derived from metastatic ccRCC. The effect of miR-146a-5p on the expression level of the five selected mRNA targets in the two cell lines is summarized in Fig 4. With the exception of BRCA1, we found significant expression changes of the other four mRNAs after miR-146a-5p treatment.

**Fig 5:**
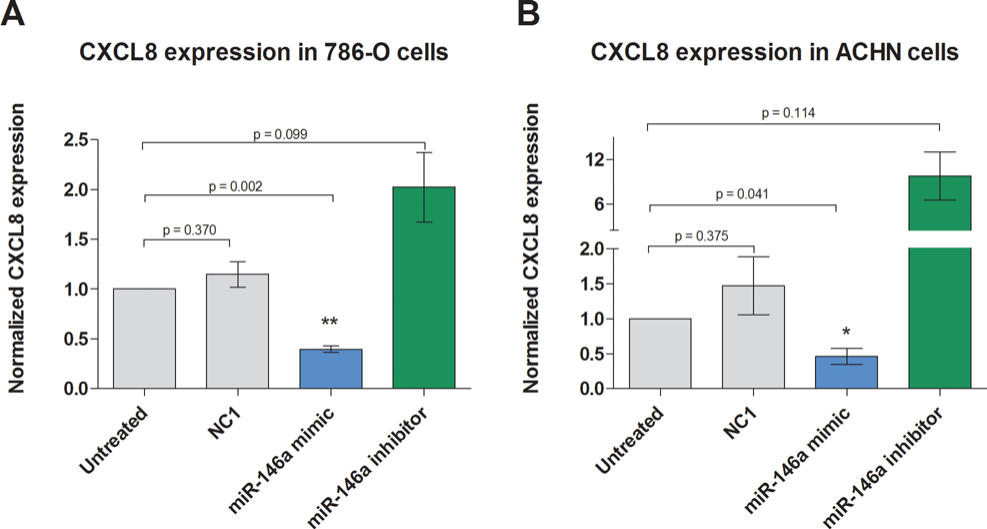
Effect of miR-146a-5p on CXCL8 protein levels in 786-O and ACHN cells. Application of miR-146a-5p mimic induced a significant reduction of CXCL8 protein expression in (A) 786-O cells and (B) ACHN cells, whereas a miR-146a-5p inhibitor rescued the CXCL8 expression levels in both cell lines. NC1 = negative control.

**Fig 6:**
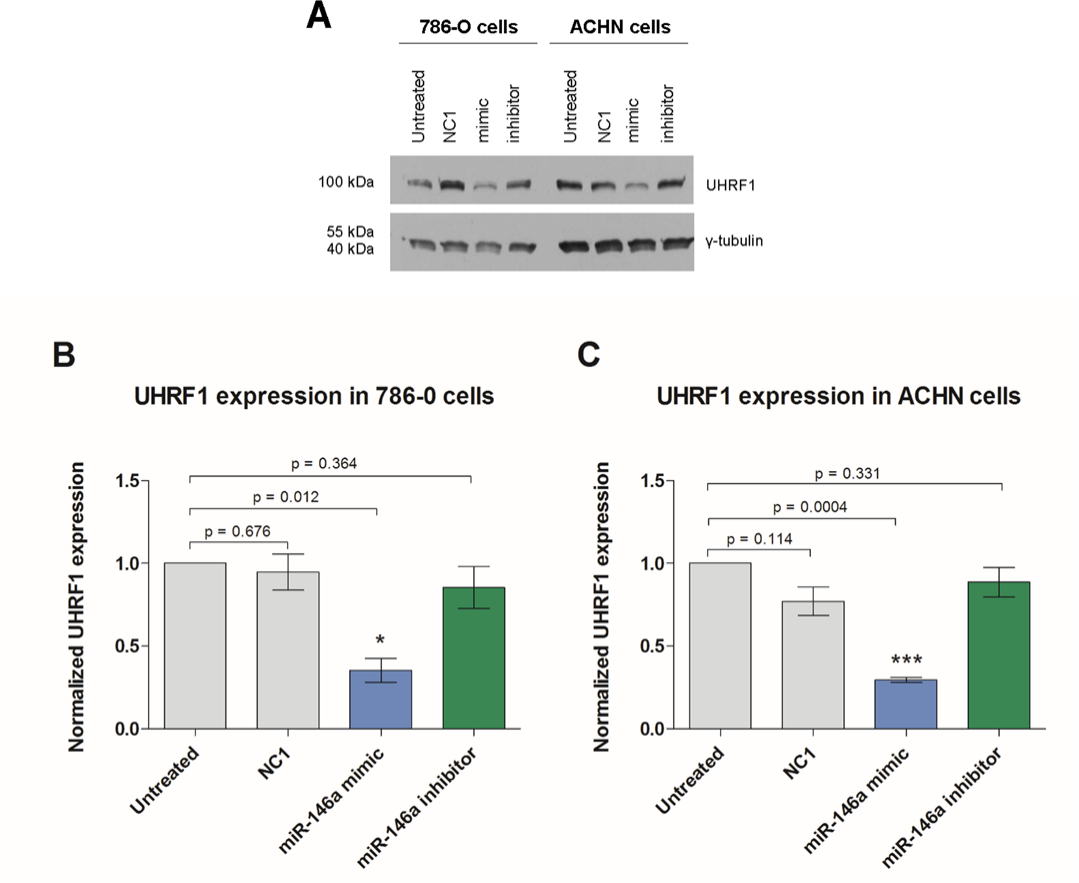
Effect of miR-146a-5p on UHRF1 protein levels in 786-O and ACHN cells. (A) Western blots and (B, C) their densitometric analyses showed a reduced UHRF1 protein expression in 786-O and ACHN cells after application of the miR-146a-5p mimic, whereas the miR-146a-5p inhibitor had no effect on UHRF1 expression levels in both cell lines. NC1 = negative control.

To confirm this regulatory effect also on the protein level, we exemplarily analyzed the protein expression of CXCL8/IL8 and UHRF1 in miRNA transfection experiments. The transfection with miR-146a-5p significantly reduced the concentration of secreted CXCL8/IL8 protein from both cell lines whereas the inhibition of miR-146a-5p increased the amount of secreted CXCL8/IL8 measured by ELISA (Fig 5). The intracellular level of UHRF1 protein was detected by Western blotting. Transfection with miR-146a-5p significantly reduced the amount of UHRF1 in both cell lines, however, a treatment with miR-146a-5p inhibitor showed no effect on UHRF1 expression (Fig 6). This missing effect was probably caused by the already low miR-146-5p levels.

## Discussion

Recent evidences indicated that miRNAs play an important role in the modulation of metastatic processes in solid tumors [24]. In case of RCC, a lot of miRNA expression profilings were performed in the last years to identify diagnostic and prognostic miRNA patterns or potential therapeutic targets [25–31]. A few studies also report on RCC metastasis related miRNA deregulation [14,32–37]. However, the knowledge about the transition from primary non-metastatic ccRCC into metastatic ccRCC is still limited. In this respect, the integrated evaluation of expression data based on new bioinformatic algorithms can meet these challenges [9–12].

Here we analyzed miRNA and mRNA microarray expression profiles derived from primary ccRCC tumors from patients with (M1) or without (M0) diagnosed metastasis to get a better understanding of the tumor progression process. We identified anti-correlated miRNA-mRNA interaction during the process of tumor transition. By using our described IPA^®^ filter settings, we obtained a network of miRNA-mRNA interaction pairs that showed an upregulation of specific miRNAs in primary tumors in ccRCC-M0 patients and a downregulation in primary tumors from ccRCC-M1 patients. The corresponding target mRNAs were accordingly low expressed in primary tumors from M0 patients and upregulated in primary ccRCCs from M1 patients (Fig 1). In detail, the filter settings identified three miRNAs (miR-146a-5p, miR-128a-3p, and miR-17-5p), which showed a higher expression in tumors without metastasis than in tumors with metastasis. The subsequent experimental validation of the microarray data analysis reduced the selection from these three miRNAs to only one miRNA (miR-146a-5p; Table 1) and from its potential 32 mRNA targets to only five (BRCA1, MCM10, CDKN3, CXCL8, and UHRF1) (Fig 1, S3 Fig).

miR-146a-5p has already been described to be dysregulated in other types of tumors [38–46], indicating an important role of this miRNA in carcinogenesis. But it is necessary to point out that the regulation of microRNA is cancer- and target-specific. A diverse behavior in different types of cancer has been described. For example, in prostate and gastric cancers, miR-146a was upegulated and identified as tumor suppressor [40,43] whereas it was downregulated and described as an oncogen in thyroid and cervical cancers [44–46]. However, this miRNA as well as miR-146b-5p that shares the same seed sequence (GAGAACU) were not described as dysregulated miRNA in any of the ccRCC profiling studies mentioned above. There were only two RCC studies that reported on the discrete upregulation of miR-146a-5p [47,48], but no association to metastasis was found. Our study demonstrated the same upregulation in the primary tumors (Table 1), but the data additionally complemented this analysis by showing that miR-146a-5p was downregulated when transition between primary tumors and metastasis aroused. As the most profiling studies only use a criterion of at least two-fold decreased/increased expression as relevant result it can be assumed that the discretely altered expression of miR-146a-5p in RCC has been largely ignored until now. On the other site, the usefulness of our approach is evident since the facility is improved to discover novel and complex cellular networks on the basis of anti-correlated expression of miRNA and mRNA using the Ingenuity pathway analysis microRNA Target Filter.

The network described in our study displays downregulation of miR-146a-5p in primary ccRCC of M1 patients to be associated with a significant upregulation of the target mRNAs BRCA1, MCM10, CDKN3, CXCL8, and UHRF1 (Fig 3). It has been taken into account, that all these mRNAs were also upregulated in ccRCC-M0 compared with normal kidney tissue (Fig 4). Nevertheless, we assume that ccRCC-M0 is the initial state to understand the mechanisms of metastatic transition. By using our filter settings, we were able to determine the differential expression of both miRNAs and mRNAs between M0 and M1 primary tumors (Figs 2 and 3).

Our further functional analysis was mainly focused on the two potential targets CXCL8 and UHRF1 of miR-146a-5p both on the mRNA and protein level. The bioinformatic data obtained by the IPA^®^ software was proven by transfection experiments in the two typical ccRCC cell lines 786-O and ACHN. In the sense of gain-of-function experiments, increased miR-146a-5p significantly reduced the concentration of secreted CXCL8 protein into cell culture medium as well as intracellular UHRF1 protein (Fig 4).

CXCL8 is a proinflammatory chemokine involved in the neutrophils chemotaxis after infection. Its role in cancer progression has been well documented [49]. Elevated expression of CXCL8 correlates with angiogenesis and VEGF expression in endothelial cells, an increase of proliferation and survival, an increase of migration of cancer cells, an induction of neutrophils on the tumor site, with tumorigenesis and metastasis in vivo models [50]. Especially the recent study from Huang and coworkers underlines the impact of CXCL8 on hepatocellular carcinoma metastasis formation in mice via increased FOXC1 expression [51]. An inflammatory microenvironment involving also CXCL8 seems to play an important role in hepatocellular carcinoma and its metastasis formation in general [52].

CXCL8 was also found to be linked to renal tumorigenesis. Microarray profiling analysis revealed an association of *CXCL8* gene expression and renal carcinoma [53]. Moreover, *CXCL8* hypomethylation is associated with genomic instability in ccRCC vs normal tissue even though no correlation with the clinicopathological status of the patients was found [54]. Recently, another study could show that variant alleles (associated with high expression) in the *CXCL8* gene are associated with poorer survival outcome in patients with RCC who received angiogenesis inhibitors [55]. Similarly, serum CXCL8 protein levels have been correlated with tumor burden in RCC patients [56]. Interestingly, an association of CXCL8 and miR-146a-5p was found in breast cancer cells and senescent human fibroblasts [57,58]. Bhaumik et al. demonstrated that miR-146a/b-5p, when expressed in a highly metastatic human breast cell line, negatively regulated the NFκB activity. This impaired NFκB activity resulted, among others, in a reduced secreted CXCL8 into the cell culture mediums [57]. The same group could further show the ectopic expressed miR-146a/b-5p indirectly suppressed IL6 and CXCL8 secretion in primary human fibroblasts [58], suggesting that miR-146a-5p and CXCL8 might share an inflammatory signaling pathway. Our results may suggest that inflammation may be relevant also in ccRCC [56].

The other studied target of miR-146a-5p, UHRF1, plays a role in cell cycle progression and is required for tumor cell growth [59,60], migration and metastasis [61]. Knockdown of UHRF1 expression in cancer cells suppressed cell growth significantly, and the overexpression of UHRF1 promoted the proliferation of breast cancer cell lines by inducing apoptosis inhibition and angiogenesis [62]. UHRF1 acts as transcriptional repressor for the TP53 signaling pathway, by binding to specific gene promoters. UHRF1 epigenetically regulates transcription by coordinating with histone deacetylase 1 [63], by binding to methylated histones (H3K9) and by promoting histone ubiquitination, DNA condensation, and suppression of tumor suppressor genes (*CDKN2A*, *CDKN1A*, *RB1*, *MLH1*, and *PML*). UHRF1 was found to be overexpressed in many cancers, among them breast, colorectal, gastric cancer, and urinary bladder cancer (as reviewed in [64]). Data of upregulated UHRF1 expression in ccRCC was recently published after we had finished our experiments for this study [65]. The results both at mRNA and protein levels confirm our findings.

The upregulation of UHRF1 observed in the transition from primary tumors to metastasis in this study indicates that epigenetic modifications are probably intervening in this phenomenon and that miR-146a-5p is an important player in the regulation of the metastatic process in RCC. Indeed, studies have shown that UHRF1 within a macromolecular protein complex promoted the ubiquitination and the degradation of DNA methyltransferase 1 and that it insured the epigenetic inheritance by assuring the maintenance of DNA methylation in African green monkey kidney fibroblast-like cell line [66–68]. Interestingly, the miR-146a-UHRF1 regulation has been shown as a key event in the metastatic progression in gastric cancer [69]. This demonstrates that the relation between miR-146a-5p and UHRF1 may be a key axis in metastasis across carcinomas, including ccRCC as shown for the first time in our study. These data also indicates that UHRF1 might be a potential therapeutic target in which the downregulation/inhibition would help to decrease some of the biological processes involved in the metastatic progression as it was shown recently for esophageal squamous cell carcinoma [70] or increased sensitivity of chemotherapeutic target as shown in breast cancer [71].

Associations between BRCA1, MCM1, and CDKN3 as the other three potential targets of miR-146a-5p and tumorigenesis were already reported for other cancers. These targets should be only briefly characterized here, although we did not studied their relationship to ccRCC in such detail as in case of CXCL8 and UHRF1.

In contrast to our RCC example, triple-negative breast cancer is generally characterized by a downregulated BRCA1 expression and an upregulated miR-146a expression. However, in this respect, converse experimental data were reported so far [38,72]. Garcia et al. [38] interpreted their results that miR-146a overexpression inactivates BRCA1 in triple-negative breast cancers whereas Fkih M'hamed et al. [72] showed that BRCA1 expression in breast cancer lines MDA-MB-231 (triple-negative cell line) and MCF7 was not affected after transfection with miR-146a. Here we could also not approve an impact of miR-146a-5p on BRCA1 expression. Such an experimental failure to confirm a predicted target is not uncommon. Since, there were no evidences of BRCA1 dysregulation in RCC reported in the literature, we could detect an upregulation of BRCA1 in RCC compared to normal tissue for the first time.

MCM10 as part of the family of minichromosome maintenance genes are involved in cell cycle progression by mediating the DNA replication initiation and elongation process. MCM10 is essential in eukaryotic cells since the inactivation of MCM10 blocks the initiation of chromosome replication [73,74]. High expression of MCM10 was also found associated to cervical carcinogenesis [75]. The observed upregulation of MCM10 mRNA in ccRCC tumors from M1 patients as shown in our study is in line with a higher proliferative activity of the transformed primary tumor.

CDKN3 is a cyclin-dependent kinase inhibitor that regulates CDK2 kinase activation and reduce cell cycle activity [76]. On the other hand, CDKN3 is also promoting cell proliferation by reducing the sensitivity of CDKN1A and TP53 [77]. CDKN3 promotes tumorigenesis in ovarian, colorectal, and hepatocellular cancer [78–80]. Overexpression of CDKN3 enhances also cell proliferation in renal cancer cells [81]. Therefore, the observed upregulation of CDKN3 in our ccRCC tumors from M1-patients correspond with these previous findings.

In summary, by comparing miRNA and mRNA microarray expression profiles and using for this purpose the IPA^®^'s microRNA Target Filter software from QIAGEN, we identified a network of anti-correlated miRNA-mRNA that is involved in transition from primary RCC tumors to metastasis. We found miR-146a-5p to be downregulated and MCM10, CDKN3, CXCL8, and UHRF1 to be upregulated in this process. This approach of systems biology using an integrated evaluation of expression data from different molecular analytes has allowed us to preselect possible diagnostic, prognostic, and therapeutic targets through analyses of interconnected networks and datasets. The advantage to use such an integrated approach is helpful in planning studies, discovering novel interactions between molecular components, and dealing sparingly and efficiently with experimental resources. We believe that the deeper insight into the processes taking place in the transition between the non-metastatic and metastatic primary tumor does not only allows the detection of new targets but also new biomarkers. In that case, a corresponding marker or model of combined marker in tumor tissue samples could be a helpful tool for discrimination between the mentioned different risk probabilities without the use of normal tissue. We demonstrated this concept in a recently published study on the different expression of piwi-RNAs in primary RCC samples [82]. Further studies are warranted for understanding the role of the other 27 genes.

## Supporting Information

S1 Fig Histological verification of frozen tissue samples by H&E staining. (PDF)

S2 Fig Expression stability of the reference miRNA combination (miR-28, miR-103, and miR-106a) and reference gene PPIA. (PDF)

S3 Fig miRNA network of miR-146a-5p. (DOCX)

S1 Table Primer sequences for RT-qPCR. (DOCX)

S2 Table List of the mRNA-miRNA pairs of ccRCC patients diagnosed with metastasis. (XLS)
