# Supplementary material for: Integrated microRNA and mRNA Signature Associated with the Transition from the Locally Confined to the Metastasized Clear Cell Renal Cell Carcinoma Exemplified by miR-146-5p
Source: PLoS One. 2016 Feb 9;11(2):e0148746. doi: 10.1371/journal.pone.0148746 (PMC4747468; doi:10.1371/journal.pone.0148746)
Supplement: S1 Fig — (PDF) [file pone.0148746.s001.pdf]

S1 Fig. Histological verification of frozen tissue samples by hematoxylin and eosin (H&E) staining.

Example 1

Renal specimen with tumor and tumor-free tissue. (A) The primary tumor sample with carcinoma of clear cell type adjacent to non-neoplastic kidney tissue can be histologically identified by H&E staining. Tissue quality was assessed for (B) tumor tissue and (C) tumor-free kidney tissue separately before the dissection of each other was performed.

Example 2

Verification of matched malignant and tumor-free kidney tissue samples. Separately taken samples from (A, B) macroscopically inconspicuous tissue and (C, D) tumor were assessed by H&E staining. As required, larger areas of inflammation and necrosis were microdissected for receiving optimal tumor and tumor-free samples, respectively, otherwise whole tissue were taken for further analysis.

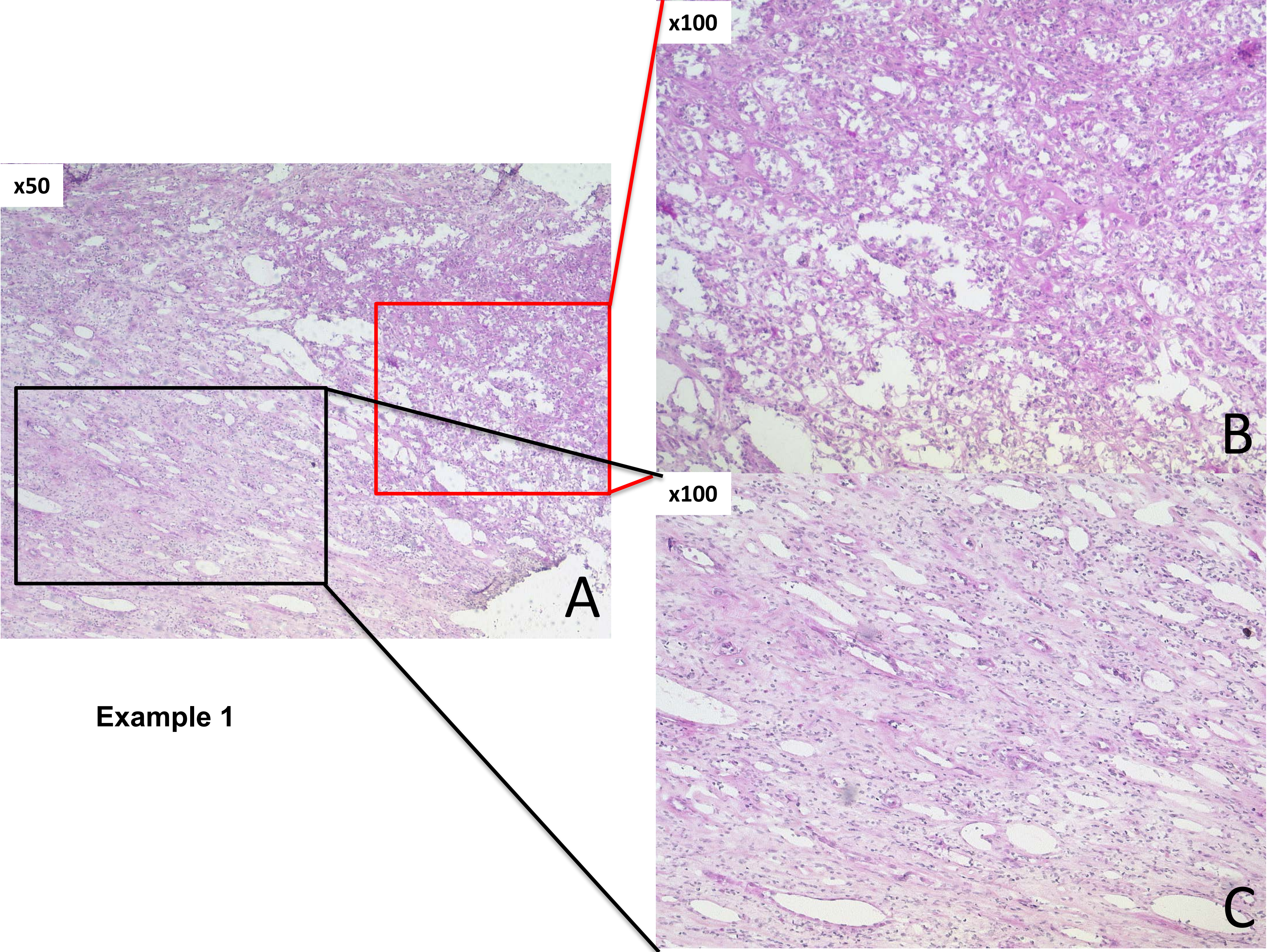

Example 1

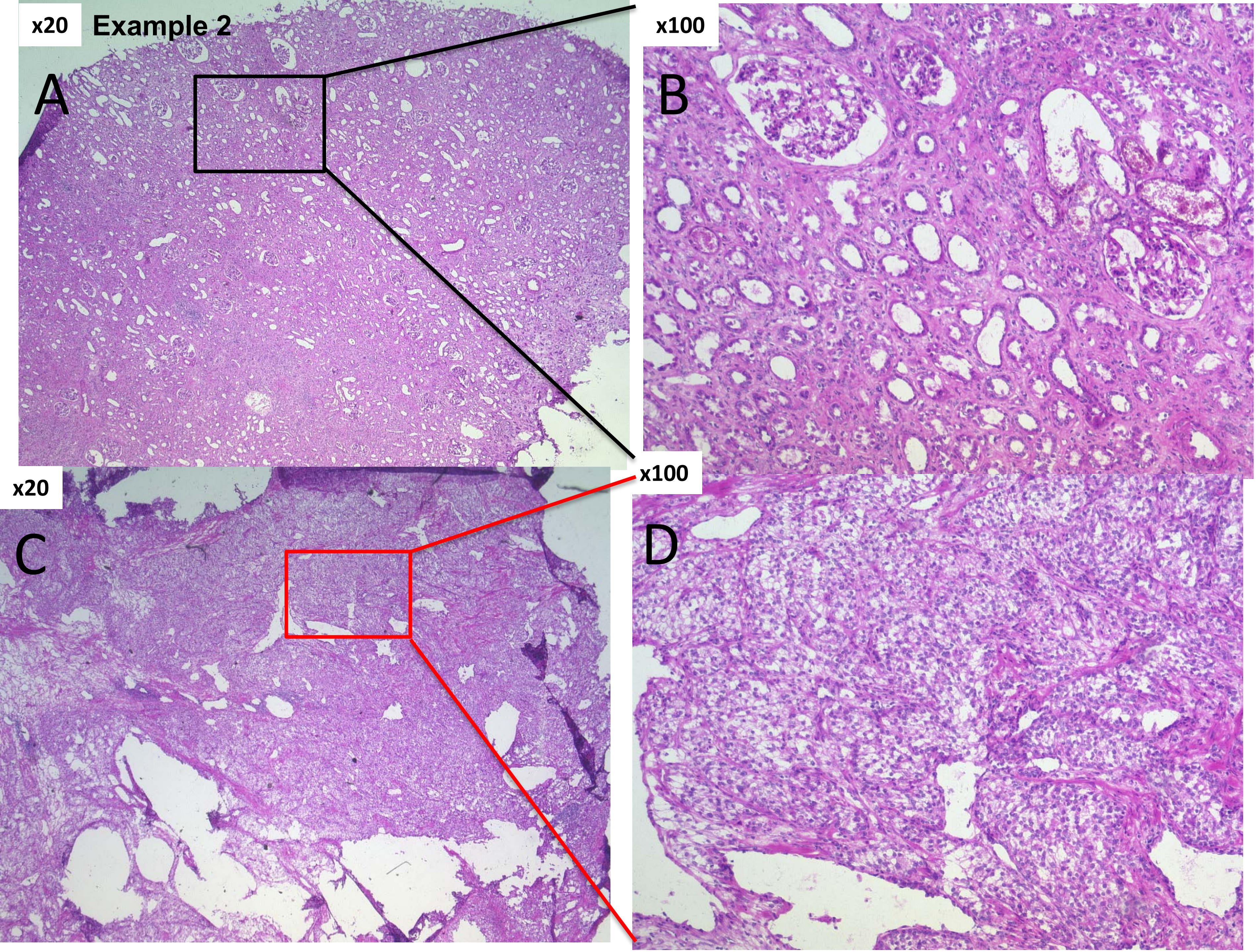
x20
Example 2
A
x100
B
x20
C
x100
D
