# Supplementary material for: Integrated microRNA and mRNA Signature Associated with the Transition from the Locally Confined to the Metastasized Clear Cell Renal Cell Carcinoma Exemplified by miR-146-5p
Source: PLoS One. 2016 Feb 9;11(2):e0148746. doi: 10.1371/journal.pone.0148746 (PMC4747468; doi:10.1371/journal.pone.0148746)

S2 Fig. Expression stability of the reference miRNA combination (miR-28, miR-103, and miR-106a) and reference gene PPIA.

We previously ascertained the combined use of miR-28, miR-103, and miR106a as suitable reference combination for the normalization of expression data in non-malignant renal tissue and malignant samples from ccRCC in order to compare the expression of miRNAs between these samples (ref. 18 in the reference list of the text). Similarly, PPIA was established as suitable reference gene to compare mRNA expression data between non-malignant and malignant tissue samples from ccRCC (ref. 19 in the reference list of the text). In this study, the suitability of these reference genes as endogenous normalizers was confirmed by their stability of expression between the examined tissue sample groups for (A) miRNAs and (B) mRNAs. Statistical significances were tested by one-way ANOVA and adjusted according to Holm-Sidak to account for multiple comparisons. Abbreviations for the tissue samples: N, "normal", non-malignant renal tissue; ccRCC-M0, tumor tissue from primary ccRCC without metastasis; ccRCC-M1, tumor tissue from primary ccRCC with metastasis.

A: Expression stability of the reference miRNA combination

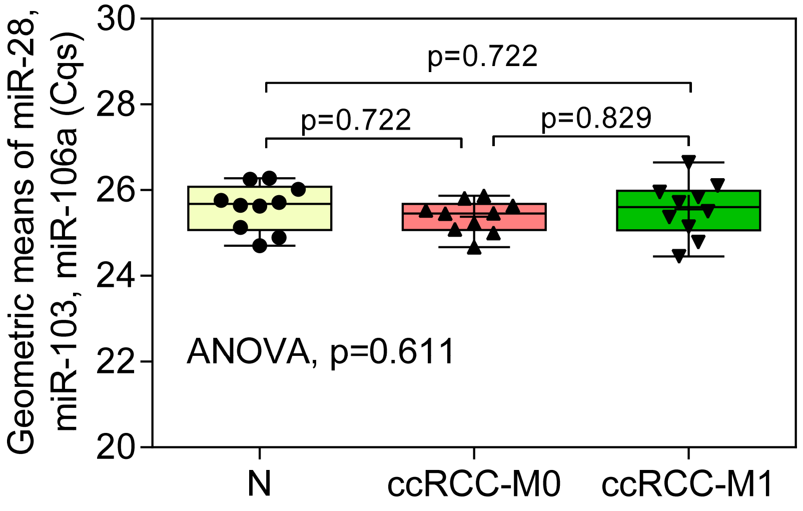

B: Expression stability of the reference gene PPIA

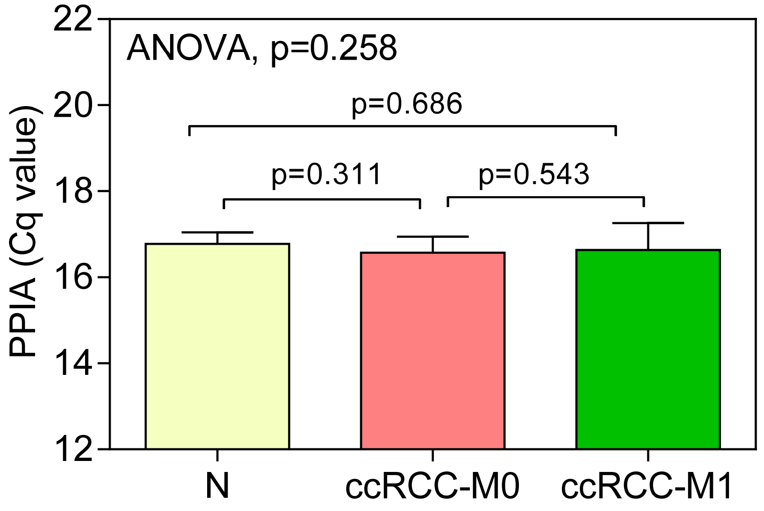

Supplement: S2 Fig — (PDF) [file pone.0148746.s002.pdf]
