# Supplementary material for: Integrated microRNA and mRNA Signature Associated with the Transition from the Locally Confined to the Metastasized Clear Cell Renal Cell Carcinoma Exemplified by miR-146-5p
Source: PLoS One. 2016 Feb 9;11(2):e0148746. doi: 10.1371/journal.pone.0148746 (PMC4747468; doi:10.1371/journal.pone.0148746)
Supplement: S3 Fig — (DOCX) [file pone.0148746.s003.docx]

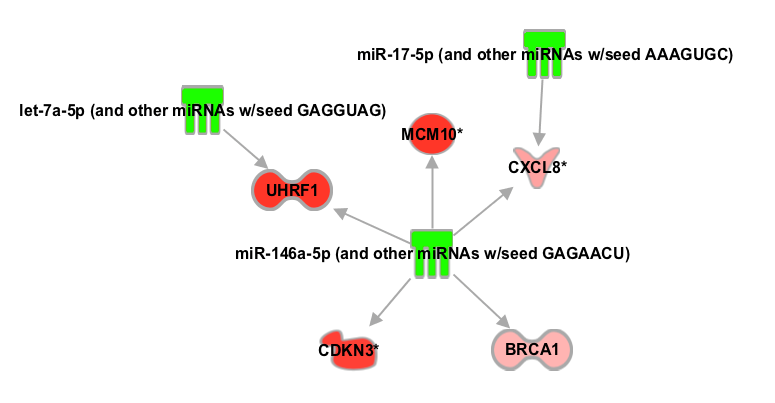


**S1 Fig. miRNA network of miR-146a-5p.** miR-146a-5p targeted network of five selected genes important for tumor progression and metastasis. miRNAs: downregulated (green) M1 vs M0; mRNAs: upregulated (red) M1 vs M0.
