# Supplementary material for: Integrated microRNA and mRNA Signature Associated with the Transition from the Locally Confined to the Metastasized Clear Cell Renal Cell Carcinoma Exemplified by miR-146-5p
Source: PLoS One. 2016 Feb 9;11(2):e0148746. doi: 10.1371/journal.pone.0148746 (PMC4747468; doi:10.1371/journal.pone.0148746)
Supplement: S1 Table — (DOCX) [file pone.0148746.s004.docx]

**S1 Table. Primer sequences for RT-qPCR.**

| **Gene symbol** | **Gene name**  **(HGNC: ID)** | **Primer/probe sequences**  **(5’---3’)** | **Amplicon**  **length (bp)** | **Temp**  **(°C)** |
| --- | --- | --- | --- | --- |
| ***CXCL8; alias IL8*** | Chemokine (C-X-C motif) ligand 8/interleukin 8  (HGNC: 6025) | F: TCTGCAGCTCTGTGTGAAGG  R: AATTTCTGTGTTGGCGCAGT | 153 | 60 |
| ***UHRF1*** | Ubiquitin-like protein containing PHD and ring finger domains 1  (HGNC: 12556) | F: AAGTGGAGGAGACGTTCCAG  R: GATCTGTCCAGGCAGTCCTT | 103 | 61 |
| ***BRCA1*** | Breast cancer 1, early onset  (HGNC: 1100) | F: ACCCCTTACCTGGAATCTGG  R: GGCAAACTTGTACACGAGCA | 196 | 57 |
| ***MCM10*** | Mini-chromosome maintenance complex component 10  (HGNC: 18043) | F: AAGAAGAAGTTCCCGCATCA  R: CTGGGCTTGCTGTCTGTTTA | 174 | 57 |
| ***CDKN3*** | Cyclin-dependent kinase inhibitor 3  (HGNC: 1791) | F: ACAGCCTGCGAGACCTAAGA  R: GCAGCTAATTTGTCCCGAAA | 88 | 57 |
| ***PPIA^a^*** | Peptidylprolyl isomerase A (cycophilin A)  (HGNC: 9253) | Hs_PPIA_1_SG QuantiTect Primer Assay (200) (QT00052311); F+R mix (Qiagen) | 121 | 55 |

^a^Reference gene for mRNA expression studies in renal cell carcinoma (see ref. 17 in the reference list).
